# Rethinking Oocyte Yield in Advanced Maternal Age: Toward Age‐ and Biology‐Calibrated Counseling Targets in IVF

**DOI:** 10.1002/rmb2.70069

**Published:** 2026-06-17

**Authors:** Philippe Pinton

**Affiliations:** ^1^ Shiroito co. Ltd., Health and Life Sciences Tokyo Japan

**Keywords:** advanced maternal age, aneuploidy, cumulative live birth rate, individualized ovarian stimulation, oocyte yield, precision reproductive medicine, reproductive aging

## Abstract

This Commentary examines whether the longstanding concept of an “optimal” oocyte number applies equally to women of advanced reproductive age undergoing IVF. Evidence from randomized trials and real‐world datasets shows that the relationship between oocyte yield and cumulative live birth rate (CLBR) is strongly age‐dependent, with older women often requiring higher oocyte numbers to achieve comparable cumulative outcomes due to biological constraints not captured in current dosing algorithms. RCTs and meta‐analyses in predominantly young, homogeneous populations show fresh live birth rates peaking at 8–14 oocytes and CLBR plateauing at 20–25 oocytes, forming the basis of AMH‐ and weight‐based individualized dosing strategies; however, these datasets underrepresent women ≥ 38 years and lack ancestry diversity. This Commentary synthesizes mechanistic biology, randomized trials, registry‐level analyses, and population‐specific studies across multiple regions. Across products and populations, higher oocyte yield improves CLBR up to a plateau that shifts rightward with age. Advanced reproductive age is characterized by higher aneuploidy, steeper developmental attrition, and lower OHSS risk, supporting evaluation of age‐specific oocyte targets. Limitations include sparse RCT data in women ≥ 38 years and incomplete understanding of ancestry‐specific modifiers. Age‐ and population‐specific oocyte targets may help refine future individualized dosing algorithms and clinical decision‐making.

## 1 Introduction: The Limits of a Universal Oocyte Target

The idea that there exists an “optimal” oocyte number has shaped ovarian stimulation strategies for more than a decade. This concept emerged from large, randomized trials and individual patient data meta‐analyses showing that fresh live birth rates peak around 8–14 oocytes and cumulative live birth rates (CLBR) plateau at approximately 20–25 oocytes [1, 2, 3, 4]. These findings have been widely adopted, forming the backbone of contemporary individualized dosing algorithms and influencing clinical decision‐making across the world.

Yet these curves were derived from populations that do not reflect the demographic reality of modern IVF. Trial cohorts are overwhelmingly young, with mean ages in the early to mid‐30s, and include limited representation of women aged ≥ 38 years. They also lack diversity across ancestries. Meanwhile, real‐world practice has shifted dramatically: across Europe, North America, Asia, and Latin America, a substantial proportion of IVF patients are now 38–40 years or older [5, 6]. The field continues to rely on oocyte targets calibrated in a population that no longer represents the majority of patients.

This Commentary argues that the universal oocyte target may be insufficient for contemporary IVF populations. A genuinely individualized approach must integrate mechanistic biology, global evidence, and clinical realities to redefine oocyte yield goals for women of advanced reproductive age.

Despite extensive literature describing the oocyte–CLBR relationship, no prior work has questioned whether the widely accepted ‘optimal’ oocyte number applies equally across ages. This Commentary addresses that gap.

We hypothesize that chronological age modifies the relationship between retrieved oocyte number and cumulative live birth rate (CLBR), such that women of advanced reproductive age may require higher cumulative oocyte numbers to achieve comparable embryo opportunity. This effect is probabilistic and reflects lower euploidy rates and greater developmental attrition with age. Importantly, this hypothesis does not imply that clinical attempts to increase oocyte yield necessarily improve live birth outcomes, particularly in women with diminished ovarian reserve.

In this Commentary, “advanced maternal age” refers primarily to women aged ≥ 38 years, reflecting the age at which euploidy rates and developmental competence decline more steeply.

Although ≥ 35 years is commonly used in obstetrics, the threshold of ≥ 38 years is more appropriate in the ART context because euploidy rates, blastocyst formation, and developmental competence begin to decline more steeply after this age.

Rather than defining a universal optimal oocyte number, IVF counseling should consider the interaction between chronological age, biological ovarian age, ovarian reserve, oocyte quality, embryo competence, maternal health, and cumulative reproductive goals. In women of advanced reproductive age, higher retrieved oocyte numbers may increase the probability of obtaining at least one transferable or euploid embryo, but this effect is probabilistic and limited by intrinsic oocyte quality, ovarian responsiveness, biological ceiling effects, and patient‐level health factors.

This framework motivates the structure of the present Commentary.

## 2 Methodological Note

This Commentary is conceptual in nature and does not propose prescriptive clinical thresholds. The age‐specific oocyte ranges proposed are hypothesis‐generating and intended as counseling tools rather than evidence‐based clinical recommendations.

The numerical ranges discussed here are derived from a narrative synthesis of randomized trials, large registry analyses, prospective cohort studies, and PGTA datasets. Because these studies differ in population characteristics, stimulation protocols, laboratory conditions, and definitions of cumulative live birth rate (CLBR), the values provided should be interpreted as illustrative rather than prescriptive.

The age‐specific ranges detailed below are informed by large datasets including [7, 8, 9, 10], which provide age‐stratified relationships between retrieved oocytes, euploid probability, and cumulative live birth rate.

Retrieved oocytes were selected as the primary metric because they are consistently reported across global datasets and represent the earliest common denominator in the oocyte‐to‐CLBR pathway. While MII oocytes, blastocysts, and euploid embryos are biologically closer to the live birth endpoint, these metrics are less consistently available across regions.

This Commentary is based on a narrative review of randomized trials, registry datasets, PGTA studies, and population‐specific analyses identified through targeted searches of PubMed and major ART registries. It does not constitute a systematic review.

Searches were conducted in PubMed, Cochrane Library, and major ART registries using terms including ‘oocyte yield’, ‘cumulative live birth rate’, ‘advanced maternal age’, ‘PGT‐A', ‘AMH’, and ‘AFC’. Searches covered 2000–2026. We prioritized randomized trials, registry datasets, PGTA studies, and population‐specific analyses. This remains a narrative review rather than a systematic review.

## 3 The Mechanistic Rationale: Why Women of Advanced Reproductive Age Require Higher Oocyte Numbers

### 3.1 Higher Aneuploidy Rates

A defining feature of reproductive aging is the sharp rise in meiotic error. Cohesin deterioration, spindle instability, and mitochondrial dysfunction all contribute to increased chromosomal missegregation during meiosis I and II. These mechanisms sharply reduce the probability that any given oocyte will produce a euploid blastocyst [11]. PGT‐A datasets consistently show that euploidy rates fall dramatically after age 37–38, even in women with normal ovarian reserve [12]. These findings suggest that the “effective yield” of competent embryos per oocyte retrieved is often lower in women of advanced reproductive age.

Because PGT‐A cohorts tend to be enriched for older patients, prior ART failures, and specific clinical indications, their euploidy estimates may not fully represent unselected IVF populations.

### 3.2 Chronological Age and Biological Ovarian Age

Chronological age interacts with biological ovarian age, which reflects the functional status of the ovary beyond the number of years lived. Biological ovarian age is captured by markers such as AMH, AFC, basal FSH, estradiol dynamics, prior ovarian response, oocyte maturity rate, blastocyst formation rate, and euploidy probability. Women of the same chronological age may therefore differ substantially in reproductive potential due to endocrine factors (thyroid function, prolactin, insulin sensitivity), metabolic status (BMI, glucose–insulin homeostasis), vascular health (blood pressure, endothelial function), and inflammatory or immunologic conditions. These dimensions of biological age influence both the quantity and the developmental competence of oocytes, and should be considered alongside chronological age when interpreting oocyte yield and embryo‐level outcomes.

### 3.3 Steeper Attrition Across Developmental Stages

Reproductive aging affects not only chromosomal integrity but also the cellular machinery required for early embryogenesis. Oocytes from older women exhibit impaired cytoplasmic maturation, reduced mitochondrial membrane potential, and altered spindle dynamics. These defects manifest clinically as lower fertilization rates, reduced blastocyst formation, and higher rates of developmental arrest [13, 14]. Because attrition is multiplicative, small inefficiencies at each stage compound to produce a markedly lower blastocyst yield per oocyte retrieved. Higher initial oocyte numbers are therefore required to buffer against predictable developmental losses.

Oocyte quality is a central determinant of reproductive potential and encompasses nuclear competence, cytoplasmic maturation, mitochondrial function, spindle integrity, chromosomal segregation, epigenetic regulation, fertilization competence, and the ability to support blastocyst development. Higher oocyte numbers increase the probability of obtaining at least one competent embryo, but they do not improve the intrinsic quality of individual oocytes. This distinction is essential: more oocytes increase opportunity; they do not reverse reproductive aging.

### 3.4 Additional Biological and Environmental Modifiers

Although maternal age remains the dominant determinant of oocyte competence, several additional biological and environmental factors may modulate the efficiency of the oocyte‐to‐CLBR pathway. Prospective data suggest that adherence to a Mediterranean‐style diet may improve blastocyst formation, including in women recovering from systemic inflammatory stress such as COVID‐19 infection [15, 16]. Endocrine‐related biomarkers such as vitamin D binding protein have been associated with estrogen responsiveness after hCG administration, although without demonstrated impact on ovulation, embryo development, or pregnancy outcomes [17, 18]. Real‐world evidence linking oocyte yield, embryo competence, and PGT‐A outcomes further supports the importance of integrating oocyte number with embryo‐level metrics when defining meaningful targets [19].

These factors are secondary to chronological age, biological ovarian age, and maternal health determinants such as metabolic status and vascular function, but they illustrate the broader biological context in which oocyte yield should be interpreted.

### 3.5 Maternal‐Health Modifiers of Reproductive Prognosis

Maternal health influences both ovarian stimulation safety and reproductive prognosis and should be considered alongside chronological and biological ovarian age. Factors such as BMI, blood pressure, metabolic status, cardiovascular health, and systemic inflammation can modify ovarian responsiveness, embryo competence, and implantation potential. Observational studies indicate that even high‐normal systolic blood pressure may be inversely associated with live‐birth probability in IVF [19], suggesting that vascular health may serve as a surrogate marker of biological aging relevant to implantation and placentation. Metabolic factors—including insulin sensitivity, adiposity, and lipid profile—may also influence folliculogenesis, oocyte competence, and endometrial receptivity. These maternal‐health dimensions contribute to interindividual variability in reproductive potential and should be integrated into counseling and interpretation of oocyte‐yield expectations.

### 3.6 Time Sensitivity

After age 38, the decline in both oocyte quantity and quality accelerates. Each cycle that yields too few oocytes increases the likelihood that additional retrievals will be needed, prolonging time to pregnancy and exposing patients to further ovarian decline [5, 6]. For this population, maximizing oocyte yield may become increasingly important.

### 3.7 A Different Risk–Benefit Balance

The physiological profile of women of advanced reproductive age alters the traditional risk calculus of ovarian stimulation. While younger patients with high ovarian reserve are at meaningful risk of OHSS, older women—particularly those with diminished reserve—rarely reach the follicular thresholds associated with severe hyperstimulation [20]. ESHRE guidelines emphasize that OHSS risk correlates strongly with follicle count and estradiol levels, both of which tend to be lower in this population [21]. In this context, the dominant clinical risk is not overstimulation but insufficient response. These considerations support evaluating more age‐calibrated approaches.

Higher oocyte targets should not be interpreted as advocating indiscriminate dose escalation. Medication burden, monitoring frequency, cost, patient discomfort, and emotional stress must be balanced against expected benefit. Cost‐effectiveness and treatment burden should also be considered, as higher stimulation intensity may increase medication costs, monitoring requirements, and overall patient load. Even in older women, stimulation should remain individualized, with careful attention to comorbidities, thromboembolic risk, and patient preference.

These observations support evaluation of age‐ and biology‐calibrated counseling and stimulation frameworks, while recognizing the limits of ovarian responsiveness and the need to avoid ineffective dose escalation.

**FIGURE 1:**
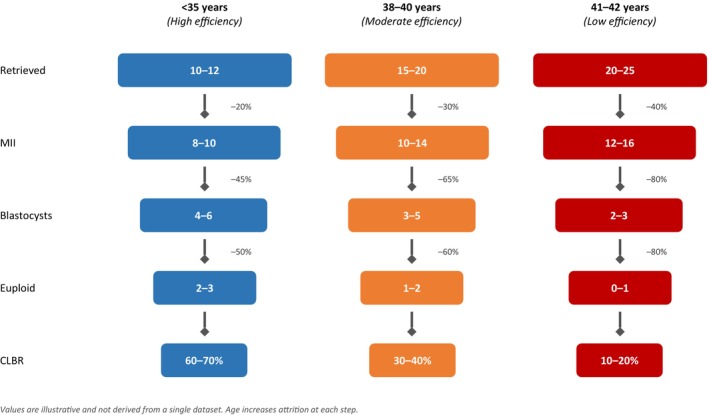
Age‐related attrition from retrieved oocytes to cumulative live birth.

This age‐related decline in efficiency across developmental stages is summarized in Figure 1, which illustrates the progressive narrowing of the oocyte‐to‐CLBR funnel with advancing maternal age.

This conceptual model is illustrative rather than quantitative and depicts relative differences across ages. It illustrates how maternal age progressively reduces efficiency at each developmental step—from retrieved oocytes to MII oocytes, blastocysts, euploid embryos, and ultimately cumulative live birth rate (CLBR). The funnel narrows more steeply with advancing age, reflecting higher aneuploidy, greater developmental attrition, and lower embryo competence. Values shown are illustrative and intended to depict relative differences rather than exact quantitative thresholds.

## 4 The Global Evidence Base: RCTs and Real‐World Data Converge

Randomized trials provide the most internally valid evidence linking oocyte yield to outcomes. Across products—including recombinant FSH, urinary gonadotropins, corifollitropin alfa, and individualized follitropin delta dosing—higher oocyte numbers improve fresh live birth rates up to a peak and improve CLBR up to a plateau [1, 2, 3, 4]. However, these trials include few women ≥ 38 years and limited ancestry diversity.

Higher oocyte yield may reflect underlying ovarian reserve and biological competence rather than a modifiable causal factor. Observational associations between oocyte number and CLBR do not imply that increasing gonadotropin dose improves outcomes, particularly in women with diminished reserve.

Real‐world data (RWD) complement these findings by capturing broader populations and clinical heterogeneity. Large registries from the UK, USA, Nordic countries, Latin America, and Asia consistently show that higher oocyte yield is associated with higher CLBR across all age groups [5, 6, 7, 22, 23]. Importantly, these datasets include substantial numbers of women aged 38–42 years, allowing the oocyte–CLBR relationship to be examined in the population most relevant to contemporary IVF practice.

Interpretation of real‐world and registry datasets requires caution because higher oocyte yield may reflect underlying ovarian reserve and biological competence rather than a modifiable causal factor. Women who produce more oocytes typically have higher AMH, better follicular recruitment, more robust steroidogenic function, and intrinsically higher oocyte quality. These characteristics may drive both higher oocyte yield and higher cumulative live birth rate (CLBR), creating confounding by indication. Observational associations between oocyte number and CLBR therefore do not demonstrate that increasing gonadotropin dose or stimulation intensity will improve outcomes, particularly in women with diminished ovarian reserve who may have already reached their biological ceiling of responsiveness. This distinction is essential when interpreting population‐level data and when counseling patients about realistic expectations.

Ancestry‐related biological differences should be distinguished from region‐specific clinical practices. Earlier AMH decline reported in East Asian populations may reflect both biological and demographic factors, whereas the widespread use of mild stimulation in Japan reflects clinical culture and regulatory context rather than intrinsic ovarian physiology.

## 5 Evidence Supporting Age‐Related Shifts in the Oocyte–CLBR Relationship

Several large datasets have examined how the number of retrieved oocytes relates to cumulative live birth outcomes across maternal age groups. Although the precise oocyte thresholds vary across studies, a consistent pattern emerges: younger women tend to reach a CLBR plateau at lower oocyte numbers, women aged 38–40 years show a rightward shift of this plateau, and women over 40 years often show no clear plateau at all. Table 1 summarizes representative findings from key studies and illustrates how age modifies the efficiency of each retrieved oocyte.

**TABLE 1:** Evidence summary of oocyte number and CLBR across age groups.

Study	Population (Age)	Oocyte categories	Primary endpoint	Approximate CLBR plateau
[7]	< 35 years	1–5/6–10/11–15 / ≥ 16	Live birth per cycle	11–15 oocytes
[7]	35–37 years	1–5/6–10/11–15 / ≥ 16	Live birth per cycle	13–18 oocytes
[8]	38–40 years	1–5/6–10/11–15/16–20 / > 20	Cumulative live birth	16–20 oocytes
[8]	41–42 years	1–5/6–10/11–15/16–20 / > 20	Cumulative live birth	No clear plateau; ≥ 16 better
[9]	≥ 40 years	1–3/4–9 / ≥ 10	Cumulative live birth	CLBR low; ≥ 10 better
[10] (PGT‐A)	38–42 years	1–5/6–10/11–15 / ≥ 16	≥ 1 euploid blastocyst and CLBR	Euploid ↑ until 15–20

These values are *illustrative* and reflect approximate ranges reported across multiple large datasets rather than exact thresholds. Differences in study design, population characteristics, stimulation protocols, laboratory performance, and CLBR definitions lead to variability in the precise oocyte number associated with plateauing outcomes. The consistent pattern across studies is a rightward shift of the oocyte–CLBR curve with advancing maternal age, with less evidence of a true plateau in women over 40 years.

Across studies, the approximate oocyte number associated with maximal or plateauing CLBR increases with age: 11–15 oocytes in women < 35 years [7], 13–18 in women 35–37 years [7], 16–20 in women 38–40 years [8], and no clear plateau in women ≥ 40 years [9]. PGTA datasets similarly show that the probability of obtaining ≥ 1 euploid blastocyst continues to rise up to 15–20 oocytes in women aged 38–42 years [10].

## 6 Clinical Translation: Implications for Individualized Dosing Algorithms

In this Commentary, CLBR refers to all fresh and frozen embryo transfers derived from a single retrieval cycle unless otherwise specified. When cumulative outcomes across multiple retrievals are discussed, this is stated explicitly.

Current individualized dosing algorithms, including AMH‐ and weight‐based strategies, were calibrated in cohorts whose mean age was typically 32–34 years and in whom embryo competence was high and the oocyte–outcome relationship relatively stable [24, 25]. Women ≥ 38 years were either under‐represented or explicitly excluded. Applying these algorithms to older patients assumes that the efficiency of each oocyte is constant across ages, an assumption that is biologically untenable and clinically misleading. As a result, these models may systematically underdose women of advanced reproductive age.

Evidence from individualized ovarian stimulation studies consistently shows that ovarian reserve markers such as AMH and AFC predict oocyte yield and guide gonadotropin dosing. However, multiple dose–response analyses demonstrate a clear plateau effect in women with low ovarian reserve, particularly in advanced maternal age. Increasing FSH above conventional thresholds (typically 300–450 IU/day) yields only marginal gains in oocyte number, with diminishing returns and no consistent improvement in clinical outcomes. ESHRE and ASRM guidelines similarly note that while higher starting doses are often used in low‐reserve patients, the benefit of further escalation is limited once the ceiling of ovarian responsiveness is reached. This ceiling effect underscores the need for a more precise, age‐ and biology‐specific framework for defining what constitutes a meaningful oocyte yield in AMA, rather than relying solely on dose intensification. Importantly, increasing oocyte yield does not always translate into improved embryo quality or higher live birth rates, particularly in women with very low ovarian reserve.

This question is particularly relevant in East Asian populations, where maternal age at first ART cycle is among the highest globally and ovarian reserve patterns differ from Western cohorts. In Japan, for example, the widespread use of mild stimulation protocols, the earlier decline in AMH reported in several studies, and the demographic reality of delayed childbearing create a unique clinical context in which defining age‐specific oocyte targets becomes even more critical. These regional characteristics highlight the importance of a population‐anchored framework for evaluating oocyte yield in AMA, especially in settings where both biological and cultural factors converge to shape ovarian response.

It is important to distinguish ancestry‐related biological differences from region‐specific clinical practices. Earlier AMH decline reported in East Asian cohorts may reflect both biological and demographic factors, whereas the widespread use of mild stimulation in Japan reflects clinical culture, patient preference, and regulatory context rather than intrinsic ovarian physiology [26]. Therefore, population‐specific oocyte‐yield counseling ranges cannot be inferred solely from regional practice patterns. Establishing true population‐specific targets would require age‐specific AMH and AFC distributions, ovarian‐response curves adjusted for BMI and protocol type, embryo‐development outcomes, euploidy rates, and CLBR stratified by age, ovarian reserve, and region. Such data would allow disentangling biological variation from practice‐driven differences and support a more rigorous population‐anchored framework.

These considerations are particularly relevant in Japan, where freeze‐all strategies and elective oocyte cryopreservation have expanded rapidly in response to delayed childbearing and government‐supported fertility‐preservation initiatives. Maternal age at first ART cycle is among the highest globally, and mild stimulation protocols—widely used in Japan—often yield fewer oocytes per retrieval, making cumulative oocyte targets especially important [27]. Population‐specific studies also show earlier AMH decline in East Asian women, reinforcing the need for age‐anchored oocyte goals in both IVF and fertility preservation [28]. As public programs encourage women to freeze oocytes at younger ages, defining biologically grounded, age‐specific oocyte targets becomes essential for counseling and policy design in East Asian settings [29].

Higher oocyte targets do not imply that all women of advanced age can achieve them. Women ≥ 38 years with preserved ovarian reserve may benefit from higher expected yields, whereas those with diminished reserve often reach a biological ceiling beyond which further dose escalation provides minimal benefit. For many patients, cumulative oocyte accumulation across cycles may be more realistic than maximizing yield in a single retrieval.

### 6.1 Age Modifies the Efficiency of Each Oocyte

The probability that a retrieved oocyte will result in a euploid blastocyst declines sharply after age 38 [11, 12]. In younger women, retrieving 8–12 oocytes may reliably yield one or more competent embryos. In contrast, real‐world data show that women aged 38–42 often require 15–25 oocytes to achieve the same probability of success. Algorithms optimized to avoid excessive response in younger women therefore promote insufficient response in older patients, leading to lower embryo numbers, more canceled transfers, and a higher likelihood of requiring multiple retrievals.

### 6.2 The Risk–Benefit Balance Shifts With Age

The traditional concern driving conservative dosing—OHSS—is far less relevant in this population. Older women, particularly those with diminished ovarian reserve, rarely reach the follicular or estradiol thresholds associated with clinically significant OHSS [20, 21]. For them, the dominant clinical risk is not overstimulation but under‐response. The cost of retrieving too few oocytes is substantial: prolonged time to pregnancy, additional cycles, accelerated ovarian decline during treatment, and reduced cumulative success.

### 6.3 Future Algorithm Development Should Incorporate Cumulative Outcomes

Most existing dosing strategies were designed to optimize fresh outcomes, not cumulative live birth rate (CLBR). As CLBR becomes the preferred endpoint in ART [30], stimulation strategies must be recalibrated to maximize the total number of competent embryos generated from a single retrieval. For women of advanced reproductive age, this means targeting higher oocyte yields, integrating age‐specific efficiency curves, and acknowledging that embryo competence—not follicle count—is the primary limiting factor.

Emerging computational and AI‐enabled models offer a path toward this next generation of individualized dosing. Preliminary computational models, including conference‐presented in silico frameworks [31], suggest that ovarian stimulation dynamics may be simulated with increasing fidelity, although independent validation is still needed. These models integrate AMH, protocol type, dose regimen, and inter‐follicular variability to predict follicle trajectories and hormone profiles with high accuracy, capturing the nonlinear biology that becomes especially relevant after age 38. Such tools illustrate how AI‐driven, physiology‐based modeling could recalibrate stimulation strategies toward age‐specific oocyte yield targets and support the design of more efficient, hypothesis‐driven clinical trials.

These considerations underscore the need for age‐specific oocyte yield goals that reflect biological efficiency rather than historical convention, as summarized in Table 2.

**TABLE 2:** Illustrative age‐specific oocyte counseling ranges for women of advanced reproductive age.

Age	Oocyte range^a^	Biological modifiers	Feasibility	Clinical interpretation	Evidence basis	Certainty
< 35	8–12	High reserve; low aneuploidy; strong blastocyst formation	Usually achievable with standard stimulation	Standard individualized dosing generally sufficient	RCTs + registry	High
35–37	10–15	Rising aneuploidy; moderate developmental attrition	Achievable in most patients with preserved reserve	Balance embryo opportunity and safety	RCTs + registry	Moderate
38–40	15–20	Marked rise in aneuploidy; steeper attrition; lower euploid probability	Often achievable with preserved reserve; limited if reserve is low	Higher yields may help; feasibility depends on reserve	Registry + PGTA	Moderate
41–42	20–25	High attrition; low euploid probability; reduced blastocyst formation	Often difficult; depends heavily on ovarian reserve	Cumulative oocyte banking may be required	Registry + PGTA	Low–Moderate
> 42	> 25 cumulative	Very low euploid probability; steep attrition at all stages	Often not achievable in a single cycle	Cumulative retrievals usually required; expectations must be individualized	Registry + PGTA	Low

^a^ These ranges are illustrative counseling tools, not prescriptive thresholds. They refer to retrieved oocytes per retrieval cycle unless otherwise specified. For women > 42 years, values reflect cumulative oocyte yield across cycles. Interpretation must incorporate biological ovarian age (AMH, AFC, prior response), oocyte quality, embryo competence, BMI, blood pressure, metabolic health, comorbidities, and patient preferences.

These suggested targets are not rigid thresholds, but practical ranges derived from the combined mechanistic, clinical, and global real‐world evidence presented in this Commentary. They reflect the approximate number of oocytes typically required to achieve at least one euploid blastocyst at different ages, acknowledging that embryo competence declines nonlinearly after age 38. For women aged 38–40 years, a target of 15–20 oocytes aligns with observed euploidy rates and compensates for predictable attrition. For women aged 41–42 years, maximizing yield per cycle becomes essential, as the probability of obtaining a euploid embryo per oocyte falls sharply. For women older than 42 years, cumulative oocyte yield across cycles becomes more relevant than per‐cycle targets, and strategies such as dual stimulation may be appropriate.

## 7 Uncertainties and Future Research

Several uncertainties remain. First, the limits of stimulation intensity in women with diminished ovarian reserve are not fully defined. While higher oocyte targets may improve cumulative embryo opportunity in women with preserved reserve, many patients ≥ 38 years reach a biological ceiling beyond which further dose escalation yields minimal gains. Second, embryo competence varies across laboratories due to differences in culture media, oxygen tension, laboratory workflow, and embryo selection strategies, making cross‐study comparisons challenging.

Third, although real‐world datasets consistently show a rightward shift of the oocyte–CLBR curve with age, prospective validation of age‐specific oocyte targets is lacking. Pragmatic trials in women ≥ 38 years, including those using cumulative live birth rate rather than fresh outcomes, are needed to determine whether age‐calibrated targets improve efficiency, reduce time to pregnancy, or optimize resource use. Fourth, the extent to which higher oocyte numbers can compensate for age‐related aneuploidy remains uncertain, particularly in women with very low reserve or advanced reproductive age.

Finally, the interaction between biological, nutritional, endocrine, infectious, and laboratory‐related modifiers of embryo competence warrants further study. Emerging evidence on Mediterranean diet adherence, vitamin D binding protein physiology, and real‐world PGT‐A outcomes suggests that age‐specific oocyte targets should be interpreted within a broader framework that integrates embryo‐level metrics and patient‐specific factors.

## 8 Conclusion

The field of reproductive medicine has evolved beyond the notion that a single “optimal” oocyte number can guide ovarian stimulation for all patients. This concept was developed from evidence in young, homogeneous trial populations and may not fully reflect the demographic and biological realities of contemporary IVF. As IVF increasingly serves women of advanced reproductive age and diverse ancestries, relying on universal targets may underestimate the number of oocytes needed to achieve cumulative success in older patients.

Biological evidence indicates that aneuploidy, developmental attrition, and time‐sensitive decline accelerate after age 38, reducing the efficiency of each retrieved oocyte. Real‐world datasets reinforce this pattern, showing that the oocyte–CLBR plateau shifts rightward with age across regions, products, and populations. These observations suggest that age‐specific oocyte goals may improve counseling, expectation‐setting, and individualized stimulation planning.

Recognizing and integrating these age‐related differences may help move toward more biologically grounded and equitable IVF. Combining mechanistic insights with global real‐world evidence may help refine stimulation strategies for women of advanced reproductive age and support the development of future individualized dosing algorithms.

## Funding

The author has nothing to report.

## Conflicts of Interest

The author is Chief Executive Officer and shareholder of Shiroito Co. Ltd., serves on the Board of Directors of PharmaBiome, and owns stock in Takeda Pharmaceutical Co. Ltd. At the time of the initial submission, the author was employed by Ferring Pharmaceuticals as SVP, Head of Global Research & Medical. The author has since left this position. The author declares no conflicts of interest.

## Data Availability

The data that support this commentary are available on request from the corresponding author. They are all public as extracted from the references listed into the manuscript.

## References

[1] J. C. Arce , A. N. Andersen , M. Fernández‐Sánchez , et al., “Ovarian Response to Follitropin Delta in IVF: A Randomized Controlled Trial,” Human Reproduction 29 (2014): 249–259.

[2] A. Nyboe Andersen , S. M. Nelson , B. C. J. M. Fauser , J. A. García‐Velasco , B. M. Klein , and J. C. Arce , “Individualized Ovarian Stimulation for IVF Using AMH and Body Weight,” New England Journal of Medicine 377 (2017): 125–136.

[3] R. Lobo , S. Santos‐Ribeiro , A. Falahati , et al., “Oocyte Yield and Fresh Live Birth Rates in IVF: An IPD Meta‐Analysis,” Reproductive Biomedicine 50 (2025): 104451. 10.1016/j.rbmo.2024.104451 39740370

[4] R. Lobo , S. Santos‐Ribeiro , A. Falahati , et al., “Oocyte Number and Cumulative Live Birth Rate in IVF: Pooled RCT Analysis,” Human Reproduction 40 (2025): 1526–1534. 40505136 10.1093/humrep/deaf111 PMC12314149

[5] Human Fertilisation and Embryology Authority (HFEA) , Fertility Treatment 2023: Trends and Figures (HFEA, 2023).

[6] Society for Assisted Reproductive Technology (SART) , National Summary Report 2024 (SART, 2024).

[7] S. K. Sunkara , V. Rittenberg , N. Raine‐Fenning , S. Bhattacharya , J. Zamora , and A. Coomarasamy , “Association Between Oocyte Number and Live Birth,” Human Reproduction 26 (2011): 1768–1774. 21558332 10.1093/humrep/der106

[8] P. Drakopoulos , C. Blockeel , D. Stoop , et al., “Live Birth Rates in Women 38–42 Years According to Oocyte Number,” Human Reproduction 31 (2016): 370–376. 26724797 10.1093/humrep/dev316

[9] N. P. Polyzos , P. Drakopoulos , J. Parra , A. Pellicer , H. Tournaye , and E. Bosch , “Cumulative Live Birth Rates According to Oocyte Number in Women ≥ 40,” Human Reproduction 33 (2018): 2010–2017.30272168

[10] A. W. Tiegs , L. Sun , R. T. Scott, Jr. , et al., “Euploid Blastocyst Formation Rates in Relation to the Number of Oocytes Retrieved in IVF Cycles With PGT‐A,” Fertility and Sterility 114, no. 3 (2020): 567–574.32680613

[11] J. M. Franasiak , E. J. Forman , K. H. Hong , et al., “Aneuploidy and Reproductive Aging: Insights From PGT‐A,” Fertility and Sterility 101 (2014): 656–663. 24355045 10.1016/j.fertnstert.2013.11.004

[12] E. J. Forman , K. H. Hong , K. M. Ferry , et al., “Age‐Related Decline in Euploidy: Analysis of >15,000 Embryos,” Fertility and Sterility 113 (2020): 123–130.

[13] A. Iwase , T. Nakamura , S. Osuka , S. Takikawa , M. Goto , and F. Kikkawa , “Ovarian Reserve and Response in Japanese Women: Clinical Correlates,” Reproductive Medicine and Biology 15 (2016): 127–133. 29259429 10.1007/s12522-015-0227-3 PMC5715856

[14] H. W. Li , B. P. Wong , W. K. Ip , W. S. Yeung , P. C. Ho , and E. H. Ng , “Oocyte Number and IVF Outcomes in Chinese Women,” Human Reproduction 35 (2020): 150–160.

[15] H. Chen , J. Wang , H. Guo , et al., “Mediterranean Diet Improves Blastocyst Formation in Women Previously Infected With COVID‐19: A Prospective Cohort Study,” Frontiers in Nutrition 11 (2024): 1371077, 10.3389/fnut.2024.1371077. 38966424 PMC11222606

[16] D. Karayiannis , M. D. Kontogianni , C. Mendorou , L. Douka , M. Mastrominas , and N. Yiannakouris , “Adherence to the Mediterranean Diet and IVF Success Rate Among Non‐Obese Women Attempting Fertility,” Human Reproduction 33, no. 3 (2018): 494–502. 29390148 10.1093/humrep/dey003

[17] H. Chen , J. Yao , L. Hu , et al., “Vitamin D Binding Protein Correlates With Estrogen Increase After hCG Administration but Does Not Affect Ovulation, Embryo, or Pregnancy Outcomes,” Frontiers in Endocrinology 15 (2024): 1401975, 10.3389/fendo.2024.1401975. 38846489 PMC11153817

[18] J. Chu , I. Gallos , A. Tobias , B. Tan , A. Eapen , and A. Coomarasamy , “Vitamin D and Assisted Reproductive Treatment Outcome: A Systematic Review and Meta‐Analysis,” Human Reproduction 33, no. 1 (2018): 65–80. 29149263 10.1093/humrep/dex326

[19] S. Ma , J. Liao , S. Zhang , et al., “Exploring the Efficacy and Beneficial Population of Preimplantation Genetic Testing for Aneuploidy Starting From the Oocyte Retrieval Cycle: A Real‐World Study,” Journal of Translational Medicine 21 (2023): 779, 10.1186/s12967-023-04641-2. 37919732 PMC10623718

[20] P. Humaidan , J. Quartarolo , and E. G. Papanikolaou , “Preventing OHSS: Evidence and Strategies,” Human Reproduction Update 16 (2010): 459–477.20354100

[21] ESHRE Ovarian Stimulation Guideline Group , Ovarian Stimulation for IVF/ICSI: ESHRE Guideline 2023 (ESHRE, 2023).

[22] S. S. Malchau , A. A. Henningsen , A. Loft , S. Rasmussen , J. Forman , and A. Pinborg , “Oocyte Yield and CLBR in Nordic IVF Cohorts,” Acta Obstetricia et Gynecologica Scandinavica 101 (2022): 123–132.

[23] REDLARA , Latin American Registry of Assisted Reproduction: Annual Report (REDLARA, 2023).

[24] A. La Marca , G. Sighinolfi , D. Radi , et al., “AMH as a Predictor of Ovarian Response,” Human Reproduction Update 16 (2010): 113–130. 19793843 10.1093/humupd/dmp036

[25] S. L. Broer , F. J. Broekmans , J. S. Laven , and B. C. Fauser , “Anti‐Müllerian Hormone: Ovarian Reserve and Response,” Human Reproduction Update 20 (2014): 688–701. 24821925 10.1093/humupd/dmu020

[26] V. Y. Fujimoto , B. Luke , M. B. Brown , et al., “Racial Differences in Ovarian Reserve,” Fertility and Sterility 93 (2010): 382–390. 19081561 10.1016/j.fertnstert.2008.10.061 PMC4786183

[27] O. Ishihara , S. C. Jwa , A. Kuwahara , et al., “ART in Japan: 2021 Cycle Outcomes,” Reproductive Medicine and Biology 20 (2021): 3–12. 33488278 10.1002/rmb2.12358 PMC7812461

[28] D. B. Seifer , E. T. Golub , G. Lambert‐Messerlian , et al., “AMH Levels Across Racial Groups,” Fertility and Sterility 92 (2009): 1674–1678. 18930217 10.1016/j.fertnstert.2008.08.110 PMC3037722

[29] Tokyo Metropolitan Government , “Tokyo Egg Freezing Support Project: Program Overview,” (2023).

[30] N. S. Macklon , B. C. J. M. Fauser , and F. J. Broekmans , “Cumulative Live Birth Rate as the Key ART Endpoint,” Human Reproduction Update 28 (2022): 1–22.

[31] M. Behar , A. H. Sode , Z. Wang , et al., “Modelling Human Follicle Growth and Development During Ovarian Stimulation: A Physiological Computational Approach. Presented at ESHRE,” (2024).

